# Angle of Insertion and Torsional Resistance of Nickel–Titanium Rotary Instruments

**DOI:** 10.3390/ma14133744

**Published:** 2021-07-04

**Authors:** Dario Di Nardo, Alessio Zanza, Marco Seracchiani, Orlando Donfrancesco, Gianluca Gambarini, Luca Testarelli

**Affiliations:** 1 Department of Oral and Maxillo-Facial Sciences, Sapienza University of Rome, 00161 Rome, Italy; dario.dinardo@uniroma1.it (D.D.N.); ale.zanza@gmail.com (A.Z.); marco.seracchiani@uniroma1.it (M.S.); gianluca.gambarini@uniroma1.it (G.G.); luca.testarelli@uniroma1.it (L.T.); 2 Department of Anatomy, Histology, Forensic Medicine and Orthopaedics, Sapienza University of Rome, 00161 Rome, Italy

**Keywords:** access cavity, endodontic instruments, fracture resistance, torsional fracture

## Abstract

Previously published studies have investigated the influence of instrument access on cyclic fatigue resistance. However, no studies have evaluated the relationship between angulated access and torsional resistance. The aim of this study was to investigate the influence of the angle of access on the torsional resistance of endodontic instruments. One hundred and eighty instruments were selected: 90 F-One Blue 25/04 and 90 HeroShaper 25/04 instruments. Three subgroups (*n* = 30) for each instrument type (A and B) were established according to the angle of insertion of the instruments inside the artificial canal (0°, 10° and 20°). The tests were performed using a custom-made device consisting of the following: a motor that can record torque values of 0.1 s; interchangeable stainless-steel canals with different curvature (0°, 10° and 20°) that allow the instrument’s angulated insertion and keep it flexed during testing procedures; and a vise used to secure the instrument at 3 mm from the tip. Torque limit was set to 5.5 Ncm, and each instrument was rotated at 500 rpm until fracture occurred. Torque to fracture (TtF) was registered by the endodontic motor, and the fragment length (FL) was measured with a digital caliper. Fractographic analysis was performed using a scanning electron microscopy (SEM) evaluation to confirm the cause of failure. TtF values and fragment length (FL) values were statistically analyzed using one-way analysis of variance (ANOVA) test and the Bonferroni correction for multiple comparisons across the groups with significance set to a 95% confidence level. Regarding the F-One Blue instruments, the results showed a higher TtF for group A3 (20°) than for group A1 (0°) and group A2 (10°), with a statistically significant difference between group A3 and the other two groups (*p* < 0.05), whereas no statistically significant difference was found between group A1 and group A2 (*p* > 0.05). Regarding the HeroShaper instrument, the results showed the highest TtF for group B3, with a statistically significant difference between the three subgroups B1, B2 and B3 (*p* < 0.05). The results showed that the torsional resistance increases as the angle of instrument access increases with a varying intensity, according to the crystallographic phase of the instrument selected.

## 1. Introduction

As demonstrated by previous studies, the intracanal separation of nickel–titanium rotary instruments could influence the short- and long-term outcome of endodontic therapies, and this is considered a major concern for general dentists and endodontists [1]. This can occur because the instrument fragment could limit shaping and disinfection procedures, increasing the possibility of leaving bacteria inside the root canal system [2]. Even when the fragment is removed, the long-term prognosis is affected, because the fragment’s removal could result in a more demolitive procedure, which could compromise the root canal wall thickness and the tooth resistance [1,3].

Fortunately, instrument separation incidence rate is not too frequent, since it has been reported that the range for stainless steel instruments is between 0.25% and 6%, whereas the range for NiTi rotary instruments is between 1.3% and 10.0% [1,4,5]. As demonstrated by Sattapan et al., the main causes of intracanal rotary instruments’ failure arise from an excessive flexural and/or torsional stress and cyclic fatigue that overcome the plastic limit of nickel–titanium alloy [6]. Regarding this, torsional failure occurs when the tip of the endodontic rotary instrument remains blocked between dentinal walls and its coronal part continues to rotate [7,8].

Over the years, in order to reduce the incidence of intracanal separation due to torsional load, manufacturers have improved the instruments’ mechanical features, such as type of alloy, thermal treatments, motion and geometric design (e.g., cross-section, instrument length, taper, pitch and rake angle), but sudden fracture can still occur [9,10,11,12,13].

The long-term outcome of endodontic therapies has been demonstrated to be influenced by dentinal tooth loss as well as by chemo-mechanical disinfection and obturation quality [14,15]. The fracture strength of endodontically treated teeth principally arises from dentinal loss due to root canal shaping and access cavity extent, as well as from prosthetic rehabilitation procedures. Regarding root canal instrumentation, it has been reported that as the canal taper and diameter of the coronal preparation increase, the fracture susceptibility of the tooth also increases [16]. Despite this, Wang et al. demonstrated with a finite element analysis that the crucial anatomical regions in terms of biomechanics and fracture resistance of endodontically treated teeth are the cervical third and the root furcation, because they significantly influence stress distribution through dentinal tissues. Conservative endodontic access cavities are able to preserve such crucial dentine compared with traditional access cavities [17,18].

The difference between traditional access cavities and conservative and/or ultraconservative ones is fundamentally based on the obtainment of a straight-line access to the middle third of the root canal, without the sacrifice of a larger amount of sound dentine. Traditional access cavity can result in an unnecessary removal of dental hard tissue from the chamber roof and peri-cervical region, reducing the fracture strength of teeth [19]. On the other hand, minimally invasive approaches, despite saving dental tissues, influence the insertion angle of endodontic instruments, increasing flexural stress and reducing their cyclic fatigue resistance [19]. In a recent study, Pedullà et al. simulated the influence of the angle of file access on the cyclic fatigue resistance of endodontic rotary instruments, showing that their inclined insertion to the endodontic canal system decreases their cyclic fatigue resistance [20].

Despite this, there have been no studies to date that investigate the relationship between angled insertion of endodontic rotary instruments and their behavior under torsional stresses. As such, the aim of this research was to investigate whether the angle of insertion could influence the torsional resistance to fracture of an endodontic instrument. The null hypothesis was that the torsional resistance of an endodontic instrument does not vary between straight-line access (angle access = 0°) and angled accesses (10° and 20°).

## 2. Materials and Methods

One hundred and eighty new instruments were selected for the study: 90 F-One Blue 25/04 (Fanta, Shanghai, China) and 90 HeroShaper 25/04 (Micro-Mega, Besançon, France) instruments. These instruments were chosen because they share the tip diameter #25 and the taper 0.04 but differ in their crystallographic phase at room temperature: F-One Blue 25/04 is martensitic, whilst HeroShaper 25/04 is austenitic. The use of instruments with the same tip diameter and taper is fundamental to standardize the testing device as explained below.

Before submission to a static torsional test, each instrument was inspected and measured under a stereomicroscope (Carl Zeiss Microimaging, Göttingen, Germany) at 20× magnification to detect any sign of deformation, and none of them were discarded.

Instruments were divided into two groups according to the endodontic rotary instrument systems (A for F-One Blue and B for HeroShaper) and then into three subgroups according to the three different angulation accesses, which simulate different instrument insertions: straight-line access (subgroup 1), 10°-angle access (subgroup 2) and 20°-angle access (subgroup 3).

### 2.1. Static Torsional Test with Different Angle of Insertion

**Figure 1:**
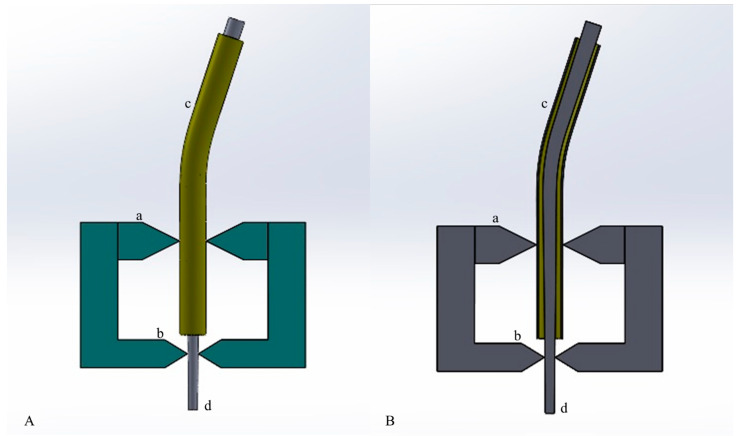
Schematic representation of the custom-made support apparatus simulating angulated access of NiTi instruments used in the static torsional test: (**A**) the support apparatus in its entirety, (**B**) its sectional view. (a) The upper part of the vise can fix the interchangeable artificial canal (20°-angled in figure); (b) the inferior part of the wise can firmly secure the instrument at 3 mm from its tip; (c) the artificial stainless-steel canal with predetermined curvature; (d) schematic representation of the NiTi instrument. The upper portion of the instrument is connected to the endodontic motor.

The static torsional tests were performed using a custom-made device. This device was composed of an electric motor and a 1:1 handpiece that was able to record torque values every 0.1 s with a precision of 0.05 Ncm (Kavo, Biberach, Germany). It was also composed of a support apparatus that was able to firmly secure the instrument at 3 mm from the tip and guide the instrument insertion at the determined angle, assuring reproducibility. The support apparatus was composed of a vise and an interchangeable stainless-steel canal simulator with a pre-determined curvature degree (0°, 10° and 20°), allowing instrument angulated insertion (Figure 1). Instruments were rotated at a constant speed of 500 rpm and a torque limit of 5.5 Ncm until fracture occurrence. Torque to fracture (TtF) was recorded for each instrument by the electric motor.

### 2.2. Fragment Length Measurement

In order to evaluate the quality of the performed tests, the fragment length of the fractured instruments was measured with a digital caliper (0.01 mm of sensitivity) considering the length from the tip of the fractured instrument to the fractured surface on the other extremity.

### 2.3. Statistical Analysis

TtF values and fragment length (FL) values were statistically analyzed using one-way analysis of variance (ANOVA) test and the Bonferroni correction for multiple comparisons across the groups with significance set to a 95% confidence level.

### 2.4. Scanning Electron Microscopy Observation

In order to evaluate the topographic features of the surface and confirm the cause of fracture, five NiTi rotary instruments fragments for each subgroup were randomly selected and then observed under a scanning electron microscopy (SEM) (Hitachi High-Technologies Corporation, Tokyo, Japan).

All of the fractured surfaces of each subgroup were examined at ×250, ×500 and ×1000 magnification for cross-sectional observation and at ×25, ×50 and ×200 magnification for the axial observation for the F-One Blue, using an accelerating voltage set between 3.00 kV and 4.00 kV and a working distance ranging from 6.0 mm to 27.4 mm according to the focus.

For the F-One Blue groups, additional acquisitions were performed in order to evidence the torsion damage on the axial flat surface.

## 3. Results

All results are summarized in Table 1.

### 3.1. Torque to Fracture (TtF) with Different Angle of Insertion

According to the data, TtF of the instruments inserted in a straight canal was 0.57 ± 0.03 Ncm for F-One Blue (group A1) and 0.80 ± 0.07 for HeroShaper (group B1), and TtF of the instruments inserted with an angulation of 10° was 0.61 ± 0.02 Ncm for group A2 and 1.05 ± 0.04 for Group B2, whilst TtF of instruments inserted with an angulation of 20° was 0.78 ± 0.02 Ncm for group A3 and 1.30 ± 0.06 for group B3. For both instruments, statistical analysis pointed out a statistically significant difference between the 20°-groups and the two other groups (0° and 10°) (*p* < 0.05). No statistically significant difference was found between group A1 and group A2 (*p* > 0.05), whilst one was found between group B1 and group B2 (*p* > 0.05).

### 3.2. Fragment Length (FL) Measurement

Regarding FL, no statistically significant differences between the three A and B subgroups were found (*p* > 0.05), with mean values and standard deviations of 3.04 ± 0.2 mm, 3.35 ± 0.09 mm and 3.26 ± 0.18 mm for groups A1, A2 and A3, respectively, and of 3.10 ± 0.08 mm, 3.06 ± 0.4 mm and 3.21 ± 0.10 mm for groups B1, B2 and B3, respectively. These results evidenced the quality and the reproducibility of the test.

### 3.3. Scanning Electron Microscopy Observation and Fractographic Analysis

**Figure 2:**
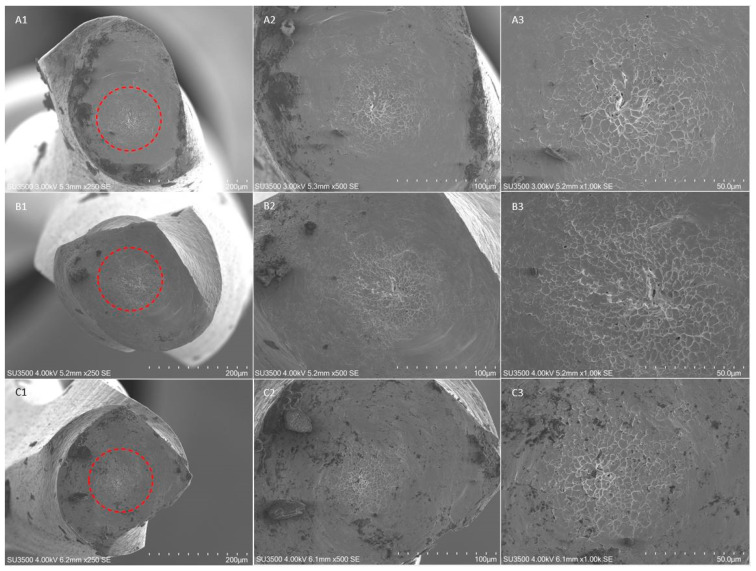
SEM images of fractured surface of three samples of F-One Blue 25/04 in a transversal view at different magnification after torsional fatigue testing. (**A1**–**A3**) Figures represent SEM images of instruments fractured after torsional test performed with a straight insertion in the artificial canal, respectively at 250×, 500× and 1000× magnification; (**B1**–**B3**) SEM images, respectively at 250×, 500× and 1000× magnification, after torsional test performed with an insertion angle of 10°; (**C1**–**C3**) SEM images, respectively at 250×, 500× and 1000× magnification, after torsional test performed with an insertion angle of 20°. The concentric circular abrasion marks and fibrous dimples near the center of rotation evidenced by the red circumferential lines are clearly represented in all samples despite the different angle of insertion.

**Figure 3:**
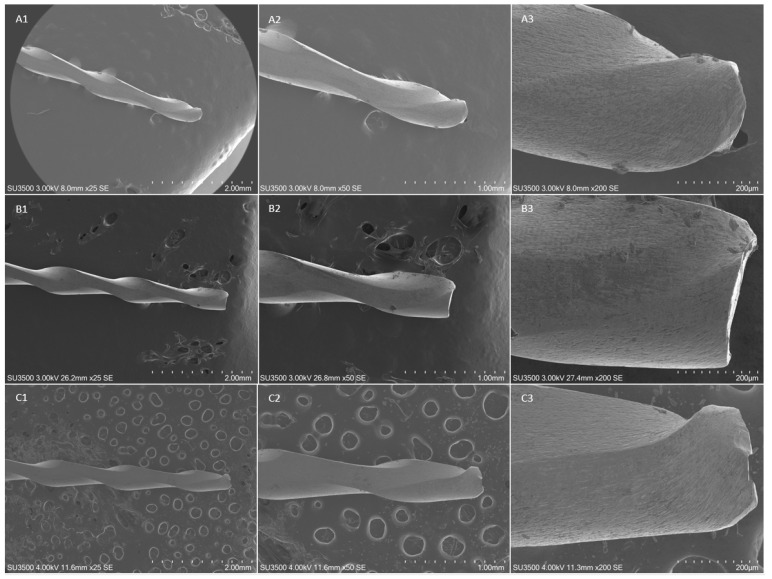
SEM images of fractured surface of three samples of F-One 25/04 in a transversal view at different magnification after torsional fatigue testing. Figures represent SEM images of instruments fractured after torsional test performed with a straight insertion in the artificial canal, respectively at 250×, 500× and 1000× magnification; (**B1**–**B3**) SEM images, respectively at 250×, 500× and 1000× magnification, after torsional test performed with an insertion angle of 10°; (**C1**–**C3**) SEM images, respectively at 250×, 500× and 1000× magnification, after torsional test performed with an insertion angle of 20°. The concentric circular abrasion marks and fibrous dimples near the center of rotation evidenced by the red circumferential lines are clearly represented in all samples despite the different angle of insertion.

**Figure 4:**
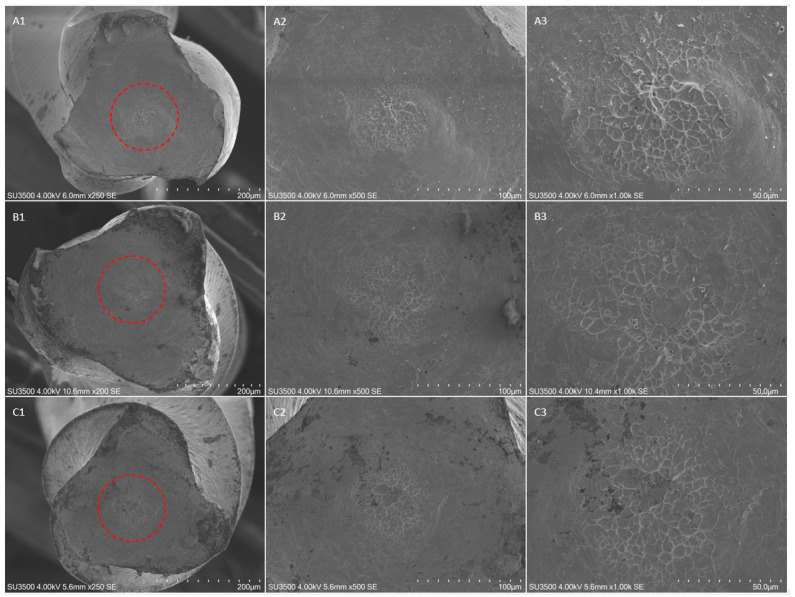
SEM images of fractured surface of three samples of HeroShaper 25/04 in a transversal view at different magnification after torsional fatigue testing. Figures represent SEM images of instruments fractured after torsional test performed with a straight insertion in the artificial canal, respectively at 250×, 500× and 1000× magnification; (**B1**–**B3**) SEM images, respectively at 200x, 500× and 1000× magnification, after torsional test performed with an insertion angle of 10°; (**C1**–**C3**) SEM images, respectively at 250×, 500× and 1000× magnification, after torsional test performed with an insertion angle of 20°. The concentric circular abrasion marks and fibrous dimples near the center of rotation evidenced by the red circumferential lines are clearly represented in all samples despite the different angle of insertion.

The fractographic analysis after SEM evaluation of the fractured fragments showed the typical features of fracture arising from excessive torsional load, showing concentric circular abrasion marks and fibrous dimples near the center of rotation, evidenced by round-shaped circumferential lines (Figure 2, Figure 3 and Figure 4).

No differences were detected in terms of fractographic analysis among the subgroups and the different insertion angles.

## 4. Discussion

Dentinal structural loss and tooth weakening are two of the greatest concerns of dentists regarding prosthetic rehabilitation and long-term success after endodontic therapies [18,21]. For this reason, different strategies have been proposed with the aim of preserving dental hard tissues, which could be reassumed in the concept of minimally invasive endodontics (MIE) [19,22,23]. As such, the extent of the access cavity and the integrity of coronal dentin, the taper of canal preparation and the preservation of the cervical region and root furcation of teeth have been thoroughly investigated [18,24]. Wang et al. stated that the taper of canals, despite not influencing the stress distribution in the canal wall, does not have a significant impact on the stress distribution of the whole tooth in comparison with the dentinal preservation of the cervical region and root furcation. In fact, those structures play a key role in dispersing functional stresses through the long axis of the tooth, ensuring its long-term stability [18].

However, the minimally invasive access cavities, such as conservative and ultra-conservative cavities, could compromise root canal instrumentation if they are not well managed [25,26]. The major complaints against constricted cavities are the inadequacy in cleaning, shaping and filling the root canal system, with an increased possibility of procedural errors, such as ledge formation, apical block or instrument failure, mostly due to a non-straight-line access of the endodontic rotary instrument to the root canal [19].

Regarding this, Pedullà et al. recently assessed that when increasing the angle of access of file, its mechanical behavior in terms of cyclic fatigue resistance decreases. The authors demonstrated that an inclined insertion could decrease cyclic fatigue resistance of NiTi rotary instruments when compared with straight-line access [20]. This occurs because when increasing the angle access, the flexural stress acting on the instrument increases.

On the contrary, the present research aimed to investigate the influence of the angled access on torsional resistance of nickel–titanium rotary instrument, because there are no data available on this theme. According to the results and the statistical analysis of this study, the null hypothesis was rejected. The 20°-angled access groups showed a statistically significant higher torque to fracture than that of the control groups (straight canals) (*p* < 0.05) for both endodontic rotary instruments selected. The F-One Blue instruments inserted in the artificial canal at an angle of 20° showed a torsional resistance 37% higher than that of the control group, whilst the HeroShaper instruments showed an increase of 63%.

The rationale behind these phenomena can be found in the relationship between the flexural stress and the torsional behavior of NiTi rotary instruments, which was first discussed by Seracchiani et al. [27]. According to this study, the registered torque to fracture of a NiTi rotary instrument can be mathematically defined as a combination of flexural and torsional stress as follows:

(1) $$M=\sqrt{M_{b}^{2}+\frac{3}{4}T^{2}}$$

*T* represents the torque, and *M_b_* is the moment of bending forces. The latter depends on the radius and degrees of curvature; the cross-sectional design; and the Young modulus, which is defined by the characteristics of the material, such as alloy and heat treatment, and, thus, it is different depending on the crystallographic phase of the instruments.

Regarding the 10°-angled access groups, no statistically significant differences were found when comparing group A2 with group A1. However, statistically significant differences were found when comparing group B2 with group B1 (with an increase in the torsional resistance of 31% for the HeroShaper inserted with an angulation of 10°). These results could have occurred because the bending moment acting on the 10°-angled F-One Blue instruments, contrary to 10°-angled HeroShaper, was not sufficient to significantly increase torsional resistance. According to this study, a higher angulation of the instrument results in a higher resistance to torsional load with different percentages in relation to the geometric characteristics and the crystallographic phase of the instruments, as evidenced by the results of this research.

The progressive increase in percentage of the torsional resistance in relation to the higher insertion angulation could have occurred because the moment of bending forces influences the operative torque exponentially rather than linearly. Thus, it is reasonable that when gradually increasing the angle of file access, the operative torque is more influenced. Further investigations are suggested to clarify the limits of the angle of access and the increase in torsional resistance according to the selection of the proper instrument in relation to its mechanical and geometrical characteristics.

F-One Blue 25/04 (Fanta, Shanghai, China) and HeroShaper 25/04 (Micro-Mega, Besançon, France) instruments were selected for this research. The first instrument is characterized by a flat design, which is demonstrated to increase cyclic fatigue resistance due to the reduced cross-sectional mass. Moreover, heat treatment (AF™-R Wire Tech, Fanta, Shanghai, China) allows them to be martensitic at room temperature [28]. As previously mentioned, heat treatment significantly influences the material’s characteristics, such as its Young modulus. Thus, different results in terms of torsional resistance were evidenced for the austenitic HeroShaper instruments when subjected to flexural moments. The latter, in fact, have a higher Young modulus than that of martensitic ones, and, thus, their bending moment is higher in the case of comparable characteristics, such as tip diameter and taper [29]. Increasing the bending moment value increases the registered torque to fracture.

Moreover, in addition to the crystallographic phase, in the computing of the different increase in terms of torsional resistance, the cross-sectional design plays a key role [30]. F-One Blue instruments have a flat S-shaped cross-section, while HeroShaper instruments have a triple helix cross-section, and, according to the above-mentioned formula, the geometric features play a crucial role in determining the bending moment acting on a flexed instrument.

Thus, the different crystallographic phase and the different geometric characteristics can explain the different percentage increases in torsional resistance.

The findings of this research, with the results obtained by Pedullà et al., could clarify, in terms of torsional and cyclic fatigue resistance, the mechanical behavior of an endodontic instrument when it is inserted in a root canal with a given angulation [20]. Since cyclic fatigue resistance is negatively affected by the angle of file access, whilst the torsional resistance is improved by it, it is reasonable to suggest the use of martensitic NiTi instruments when conservative access cavities are performed, because martensitic alloys better withstand cyclic fatigue due to their ductility, flexibility and plasticity. While torsional resistance is improved by the bending moment acting on the instruments during their flexed insertion, both cyclic fatigue and torsional resistance are thoroughly influenced by the mechanical (alloy or heat-treatment) and geometric (pitch, cross-sectional design, taper etc.) features of the selected instruments [30,31]. Caution should be used when generalizing the relationship between the mechanical behavior and the angle of access of NiTi rotary instruments, because an angled insertion could improve the torsional resistance to fracture. However, it will simultaneously affect the cyclic fatigue resistance.

The present study aimed to investigate the influence of the angled access on torsional resistance of nickel–titanium rotary instruments through a static torsional test; thus, it did not consider dynamic torsional stresses acting on the instrument during intra-operational dentinal cutting. Moreover, it simulated the insertion of the instrument when it reached the apical portion of the canal while the segment subjected to bending moment was in the coronal third. Since the latter is larger and heavier than the apical portion, due to its increased dimension, it can be deduced that it is subjected to a higher bending moment than a flexed apical segment of the instrument. Therefore, a lower increase in torsional resistance could be expected if the torsional test is performed by blocking the instrument tip as the apical part of the instrument engages the curvature, simulating the first phase of canal instrumentation.

In the present research, only the as-received endodontic instruments were included, but according to a study published by Loska et al., which evidenced the mechanical property changes after their prolonged clinical use, different results could be expected for clinically used instruments [32]. Moreover, in the present study, the surface treatment was not considered, so its influence on the mechanical properties of the selected instruments was not considered [33]. For these reasons, further studies are needed.

## 5. Conclusions

Within its limitation, this study investigated the relationship between angulated instrument insertion and its mechanical behavior in terms of torsional resistance. The results showed that when increasing the angle of instrument access, the torsional resistance to fracture increases with different percentages, according to the crystallographic phase and the geometric characteristics of the endodontic instruments.

In conclusion, considering the evidence present in literature regarding the relationship between cyclic fatigue and the insertion angle [20], the use of martensitic instruments could be suggested when conservative or ultra-conservative access cavities are performed, because their cyclic fatigue resistance is higher than that of austenitic ones, with the torsional resistance compensated by the flexural moment acting on the instrument.

## Figures and Tables

**Table 1:** Summary of the results of TtF and FL with mean values and standard deviations according to the different endodontic rotary instrument systems.

	F-One Blue 25/04			HeroShaper 25/04		
	Group A1 (Straight-Line Access)	Group A2 (10°-Angled Access)	Group A3 (20°-Angled Access)	Group B1 (Straight-Line Access)	Group B2 (10°-Angled Access)	Group B3 (20°-Angled Access)
TtF (Ncm)	0.57 ± 0.03	0.61 ± 0.02	0.78 ± 0.02	0.80 ± 0.07	1.05 ± 0.04	1.30 ± 0.06
Fragment Length (mm)	3.04 ± 0.2	3.35 ± 0.09	3.26 ± 0.18	3.10 ± 0.08	3.06 ± 0.4	3.21 ± 0.10

## Data Availability

Data sharing is not applicable for this article.

## References

[1] Madarati A.A., Hunter M.J., Dummer P.M. (2013). Management of intracanal separated instruments. J. Endod..

[2] Seven N., Cora S. (2019). Effectiveness of different irrigation systems in the presence of intracanal-separated file. Microsc. Res. Tech..

[3] Wohlgemuth P., Cuocolo D., Vandrangi P., Sigurdsson A. (2015). Effectiveness of the GentleWave System in Removing Separated Instruments. J. Endod..

[4] Spili P., Parashos P., Messer H.H. (2005). The impact of instrument fracture on outcome of endodontic treatment. J. Endod..

[5] Iqbal M.K., Kohli M.R., Kim J.S. (2006). A retrospective clinical study of incidence of root canal instrument separation in an endodontics graduate program: A PennEndo database study. J. Endod..

[6] Sattapan B., Nervo G.J., Palamara J.E., Messer H.H. (2000). Defects in rotary nickel-titanium files after clinical use. J. Endod..

[7] Sattapan B., Palamara J.E., Messer H.H. (2000). Torque during canal instrumentation using rotary nickel-titanium files. J. Endod..

[8] Gambarini G., Miccoli G., Di Nardo D., Del Giudice A., Mazzoni A., Seracchiani M., Testarelli L. (2020). Torsional resistance of two new heat treated nickel titanium rotary instruments: An in vitro evaluation. Pesquisa Brasileira em Odontopediatria e Clinica Integrada.

[9] Baek S.H., Lee C.J., Versluis A., Kim B.M., Lee W., Kim H.C. (2011). Comparison of torsional stiffness of nickel-titanium rotary files with different geometric characteristics. J. Endod..

[10] Zupanc J., Vahdat-Pajouh N., Schäfer E. (2018). New thermomechanically treated NiTi alloys—A review. Int. Endod. J..

[11] Gambarini G., Seracchiani M., Zanza A., Miccoli G., Del Giudice A., Testarelli L. (2021). Influence of shaft length on torsional behavior of endodontic nickel-titanium instruments. Odontology.

[12] Seracchiani M., Miccoli G., Reda R., Zanza A., Obino F.V., Bhandi S., Gambarini G. (2020). and Testarelli, L. A comprehensive in vitro comparison of mechanical properties of two rotary endodontic instruments. World J. Dent..

[13] Gambarini G., Cicconetti A., Nardo D.D., Miccoli G., Zanza A., Testarelli L., Seracchiani M. (2020). Influence of different heat treatments on torsional and cyclic fatigue resistance of nickel-titanium rotary files: A comparative study. Appl. Sci..

[14] Ng Y.L., Mann V., Rahbaran S., Lewsey J., Gulabivala K. (2007). Outcome of primary root canal treatment: Systematic review of the literature—Part 1. Effects of study characteristics on probability of success. Int. Endod. J..

[15] Ng Y.L., Mann V., Rahbaran S., Lewsey J., Gulabivala K. (2008). Outcome of primary root canal treatment: Systematic review of the literature—Part 2. Influence of clinical factors. Int. Endod. J..

[16] Munari L.S., Bowles W.R., Fok A.S.L. (2019). Relationship between Canal Enlargement and Fracture Load of Root Dentin Sections. Dent Mater..

[17] Yuan K., Niu C., Xie Q., Jiang W., Gao L., Huang Z., Ma R. (2016). Comparative evaluation of the impact of minimally invasive preparation vs. conventional straight-line preparation on tooth biomechanics: A finite element analysis. Eur. J. Oral Sci..

[18] Wang Q., Liu Y., Wang Z., Yang T., Liang Y., Gao Z., Fang C., Zhang Y. (2020). Effect of Access Cavities and Canal Enlargement on Biomechanics of Endodontically Treated Teeth: A Finite Element Analysis. J. Endod..

[19] Silva E., Pinto K., Ferreira C., Belladonna F., De-Deus G., Dummer P., Versiani M. (2020). Current status on minimal access cavity preparations: A critical analysis and a proposal for a universal nomenclature. Int. Endod. J..

[20] Pedullà E., La Rosa G.R.M., Virgillito C., Rapisarda E., Kim H.C., Generali L. (2020). Cyclic Fatigue Resistance of Nickel-titanium Rotary Instruments according to the Angle of File Access and Radius of Root Canal. J. Endod..

[21] Wolters W.J., Duncan H.F., Tomson P.L., Karim I.E., McKenna G., Dorri M., Stangvaltaite L., van der Sluis L.W.M. (2017). Minimally invasive endodontics: A new diagnostic system for assessing pulpitis and subsequent treatment needs. Int. Endod. J..

[22] Bürklein S., Schäfer E. (2015). Minimally invasive endodontics. Quintessence Int..

[23] Clark D., Khademi J. (2010). Modern molar endodontic access and directed dentin conservation. Dent. Clin. N. Am..

[24] Sabeti M., Kazem M., Dianat O., Bahrololumi N., Beglou A., Rahimipour K., Dehnavi F. (2018). Impact of Access Cavity Design and Root Canal Taper on Fracture Resistance of Endodontically Treated Teeth: An Ex Vivo Investigation. J. Endod..

[25] Patel S., Rhodes J. (2007). A practical guide to endodontic access cavity preparation in molar teeth. Br. Dent. J..

[26] Krishan R., Paqué F., Ossareh A., Kishen A., Dao T., Friedman S. (2014). Impacts of Conservative Endodontic Cavity on Root Canal Instrumentation Efficacy and Resistance to Fracture Assessed in Incisors, Premolars, and Molars. J. Endod..

[27] Seracchiani M., Miccoli G., Di Nardo D., Zanza A., Cantore M., Gambarini G., Testarelli L. (2021). Effect of Flexural Stress on Torsional Resistance of NiTi Instruments. J. Endod..

[28] Gambarini G., Miccoli G., Seracchiani M., Khrenova T., Donfrancesco O., D’Angelo M., Galli M., Di Nardo D., Testarelli L. (2019). Role of the Flat-Designed Surface in Improving the Cyclic Fatigue Resistance of Endodontic NiTi Rotary Instruments. Materials.

[29] Thompson S.A. (2000). An overview of nickel-titanium alloys used in dentistry. Int. Endod. J..

[30] Zanza A., Seracchiani M., Di Nardo D., Reda R., Gambarini G., Testarelli L. (2021). A Paradigm Shift for Torsional Stiffness of Nickel-Titanium Rotary Instruments: A Finite Element Analysis. J. Endod..

[31] Di Nardo D., Gambarini G., Seracchiani M., Mazzoni A., Zanza A., Giudice A., D’Angelo M., Testarelli L. (2020). Influence of different cross-section on cyclic fatigue resistance of two nickel-titanium rotary instruments with same heat treatment: An in vitro study. Saudi Endod. J..

[32] Loska S., Basiaga M., Pochrząst M., Łukomska-Szymańska M., Walke W., Tyrlik-Held J. (2015). Comparative characteristics of endodontic drills. Acta Bioeng. Biomech..

[33] Bumbalek M., Joska Z., Pokorny Z., Sedlak J., Majerik J., Neumann V., Klima K. (2021). Cyclic Fatigue of Dental NiTi Instruments after Plasma Nitriding. Materials.

